# Circulating microRNA-200 Family as Diagnostic Marker in Hepatocellular Carcinoma

**DOI:** 10.1371/journal.pone.0140066

**Published:** 2015-10-08

**Authors:** Sameer A. Dhayat, Anna Hüsing, Norbert Senninger, Hartmut H. Schmidt, Jörg Haier, Heiner Wolters, Iyad Kabar

**Affiliations:** 1 Department of General and Visceral Surgery, University Hospital Muenster, Muenster, Germany; 2 Department of Transplant Medicine, University Hospital Muenster, Muenster, Germany; 3 Comprehensive Cancer Center Muenster, University Hospital Muenster, Muenster, Germany; Taipei Medicine University, TAIWAN

## Abstract

**Goals:**

In this clinical study, we aimed to evaluate the role of circulating microRNA-200 family as a non-invasive tool to identify patients with cirrhosis-associated hepatocellular carcinoma (HCC).

**Background:**

Prognosis of HCC remains poor with increasing incidence worldwide, mainly related to liver cirrhosis. So far, no reliable molecular targets exist for early detection of HCC at surgically manageable stages. Recently, we identified members of the microRNA-200 family as potential diagnostic markers of cirrhosis-associated HCC in patient tissue samples. Their value as circulating biomarkers for HCC remained undefined.

**Methods:**

Blood samples and clinicopathological data of consecutive patients with liver diseases were collected prospectively. Expression of the microRNA-200 family was investigated by qRT-PCR in blood serum samples of 22 HCC patients with and without cirrhosis. Serum samples of patients with non-cancerous chronic liver cirrhosis (n = 22) and of healthy volunteers (n = 15) served as controls.

**Results:**

MicroRNA-141 and microRNA-200a were significantly downregulated in blood serum of patients with HCC compared to liver cirrhosis (*p*<0.007) and healthy controls (*p*<0.002). MicroRNA-141 and microRNA-200a could well discriminate patients with cirrhosis-associated HCC from healthy volunteers with area under the receiver-operating characteristic curve (AUC) values of 0.85 and 0.82, respectively. Additionally, both microRNAs could differentiate between HCC and non-cancerous liver cirrhosis with a fair accuracy.

**Conclusions:**

Circulating microRNA-200 family members are significantly deregulated in patients with HCC and liver cirrhosis. Further studies are necessary to confirm the diagnostic value of the microRNA-200 family as accurate serum marker for cirrhosis-associated HCC.

## Introduction

Hepatocellular carcinoma (HCC) is the major subtype of primary liver cancers and represents the fifth most common cancer and the third leading cause of cancer-related death with mortality rates reaching up to 750,000 deaths annually worldwide.[1, 2] More than 80% of HCC arise in a background of chronic liver disease with liver cirrhosis caused by chronic hepatitis B or C virus infection, alcohol abuse or obesity with non-alcoholic fatty liver disease.[3]

Current imaging and molecular marker tests are unsatisfactory, particularly for early detection of HCC at surgically manageable stages.[4, 5] Therefore, there is an urgent need to identify accurate diagnostic and therapeutic targets for HCC.

A variety of recent studies provide clear evidence that microRNAs—an emerging class of highly conserved, non-coding small RNAs that regulate gene expression at the post-transcriptional level—are abundant in the liver and can potentially regulate every aspect of cellular activity, including differentiation and development, metabolism and proliferation.[6, 7] The discovery of highly stable microRNAs circulating in blood and protected from RNAase-mediated degradation has led to increased research focus on disease-related variations in serum and plasma microRNA concentrations.[8, 9]

Recently, we could demonstrate in clinical tissue samples that members of the microRNA-200 family have potential as diagnostic markers for the detection of cirrhosis-associated HCC.[10] Further, we confirmed the regulatory effect of the microRNA-200 family as a suppressor of epithelial–mesenchymal transition and cancer cell migration through targeting the zinc finger E-box-binding transcription factor and mesenchymal marker ZEB-1. ZEB-1 and vimentin were significantly upregulated, whereas correspondingly E-cadherin was significantly downregulated in HCC compared to non-cancerous liver specimens with and without cirrhosis. Similarly, the specific role of the microRNA-200 family as potential diagnostic marker was demonstrated for other carcinoma entities, such as colorectal, breast, and endometrial cancer.[11, 12]

In this clinical study, we aimed to evaluate the role of the microRNA-200 family as circulating diagnostic marker in cirrhosis-associated HCC.

## Materials and Methods

### Patients and samples

A blood serum bank and follow-up database are maintained prospectively by the Department of General and Visceral Surgery, the Department of Transplant Medicine, and the Comprehensive Cancer Center Muenster, University Hospital Muenster, Germany. From these, 22 blood serum samples of HCC patients were collected between November 2014 and April 2015. Serum samples of 22 patients with chronic liver cirrhosis and of 15 healthy volunteers served as controls. About 5 ml of venous blood was collected from each participant by a study nurse under standardized conditions from 8 to 10 am as part of the routine ambulatory blood sample collection. The whole blood was separated into serum and cellular fractions by centrifugation at 1,200 g for 10 minutes after a recommended clotting time of minimum 30 minutes. The supernatant serum was stored at -80°C until analysis.

Our study was approved by the local ethics committee (Ethik Kommission der Ärtzekammer Westfalen-Lippe und der Medizinischen Fakultät der Westfälischen Wilhelms-Universität, Az: 1IXHai). Written informed consent was given by all participants for biospecimen collection and for recording their clinical data and use in anonymized analysis.

In addition to 4 serum samples of HCC patients without cirrhosis, serum samples of cirrhosis-associated HCC Batts and Ludwig stage 4 (n = 18) were obtained from patients with hepatitis B or C (n = 9), alcoholic liver disease (n = 6), nonalcoholic steatohepatitis (n = 1), and cirrhosis of cryptogenic origin (n = 2). Healthy serum samples were obtained from student volunteers without medical history. Serum samples of chronic liver cirrhosis Batts and Ludwig stage 4 were obtained from patients with hepatitis B or C (n = 11), alcoholic liver disease (n = 7), alpha-1 antitrypsin deficiency (n = 1), Budd-Chiari syndrome (n = 1) and cirrhosis of cryptogenic origin (n = 2).

**Fig 1:**
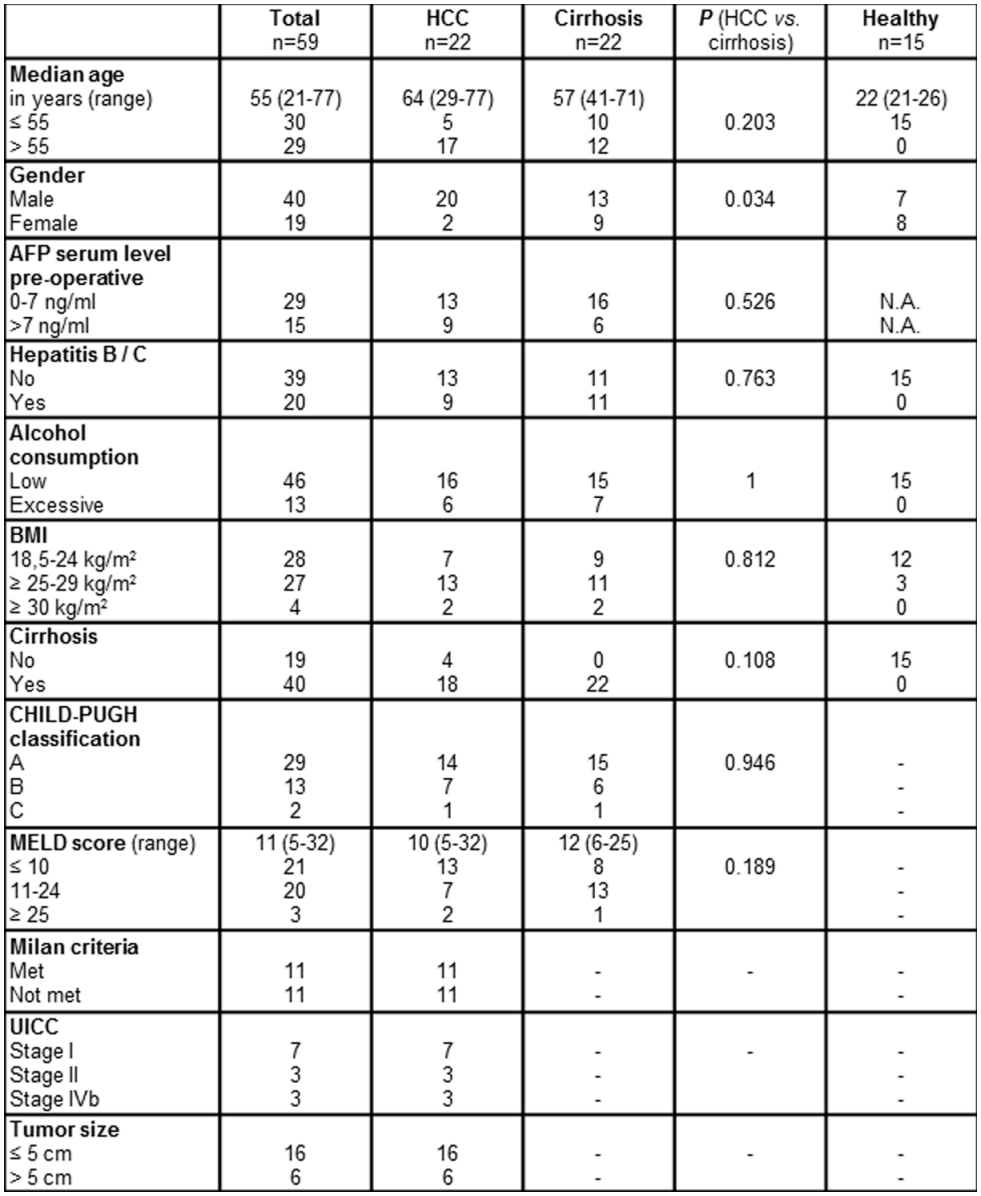
Clinicopathological characteristics of study participants. P<0.05 indicates significance. N.A.: not available.

Patients that received immunosuppression, chemo- or radiotherapy before blood sampling were excluded to avoid potential influences on microRNA expression. Perioperative clinical data, histo-pathological information and follow-up data were collected for all patients (Fig 1).

### RNA isolation and quantification of microRNA-200 family

Total RNA was isolated from 59 cryopreserved blood serum samples using QIAzol Lysis Reagent (Qiagen, Hilden, Germany) as a part of the miRNeasy Serum/Plasma Kit (Qiagen) according to the manufacturer’s instructions. 3.5 μl synthetic miRNA-39 from *Caenorhabditis elegans* (cel-microRNA-39) were added as a spike-in control (1.6 x 10^8^ copies/μl working solution). RNA concentration and purity were assessed by Agilent 2100 Bioanalyzer and RNA 6000 Nano/Pico LabChip (Agilent Tech., Boeblingen, Germany).

Quantitative Real-Time (qRT) PCR was performed using the miScript PCR system (Qiagen) as described previously.[10] Quantitative microRNA analysis was performed using CFX Manager Software v2.1 (Bio-Rad Laboratories, Munich, Germany). Expression of circulating microRNA-141, microRNA-200a, microRNA-200b, microRNA-200c, and microRNA-429 was analyzed quantitatively after normalization to the cel-microRNA-39 spiked-in control using the ΔΔCt (cycle threshold) method.[13]

### Statistical analysis

Statistical analysis was performed with the JMP Statistical Software 4.0.0 (SAS Institute, INC, Cary, NC, USA) as described previously, SPSS® Statistics Version 22 (IBM Corp. Armonk, NY), and the GraphPad Prism 6 (GraphPad Software, INC, La Jolla, CA, USA) for Windows®.[10]

Exploratory factor analysis was performed to elucidate the importance for variance in the observed variables in terms of underlying latent factors (potential coimportance of molecular biology and etiological parameters). Scree-plot and Kaiser-Meyer-Olkin (KMO-test) were used to evaluate suitability of correlation matrix. Anti-image-correlation was assumed as adequate for KMO >0.5. Principal component analysis was preferred for factorial extraction, since we aimed at available variables, but not all possible predictive parameters. Factor rotation was done for optimization of factor annotation. Additionally, linear regression analysis was used to estimate the statistical relationship between disease-related risk factors and microRNA expression.

The predicted probability of being diagnosed with HCC or non-cancerous liver cirrhosis was used as a surrogate marker to construct the receiver operating characteristic (ROC) curve. Area under the ROC curve (AUC) with its corresponding 95% confidence interval (CI) was used as an accuracy index for evaluating the diagnostic performance of the selected microRNA. Values for *p*< 0.05 were considered to be statistically significant.

## Results

### Clinicopathological characteristics of study participants

The characteristics of 22 HCC patients, 22 patients with non-cancerous liver cirrhosis and 15 healthy student volunteers were presented in Fig 1. There was no significant difference in the distribution of age, serum alpha-fetoprotein (AFP), hepatitis, alcohol abuse, body mass index (BMI), liver cirrhosis, CHILD-PUGH classification and MELD (Model for End-Stage Liver Disease) score among HCC patients and patients with non-cancerous liver cirrhosis. The MELD score was calculated using the most recent laboratory values for serum bilirubin, serum creatinine, and international normalized ratio for prothrombin time (INR). However, HCC was more prevalent in males than in females compared to gender distribution in non-cancerous cirrhotic controls (*p* = 0.034).

Moreover, we investigated tumor characteristics of HCC patients including Milan criteria, post-operative UICC stage and tumor size, which were all listed in Fig 1. 50% of HCC patients met the Milan Criteria, defined as a single HCC nodule with a maximum size of 5 cm or as many as 3 nodules with the largest not exceeding 3 cm and no macrovascular invasion. In addition to explorative laparotomies and fine needle biopsies, 10 HCC patients underwent radical resection and were assigned to atypical or anatomical partial liver resection (n = 4), hemihepatectomy (n = 1) and liver transplantation (n = 5) after blood serum collection.

### Expression of circulating microRNA-200 family members

**Fig 2:**
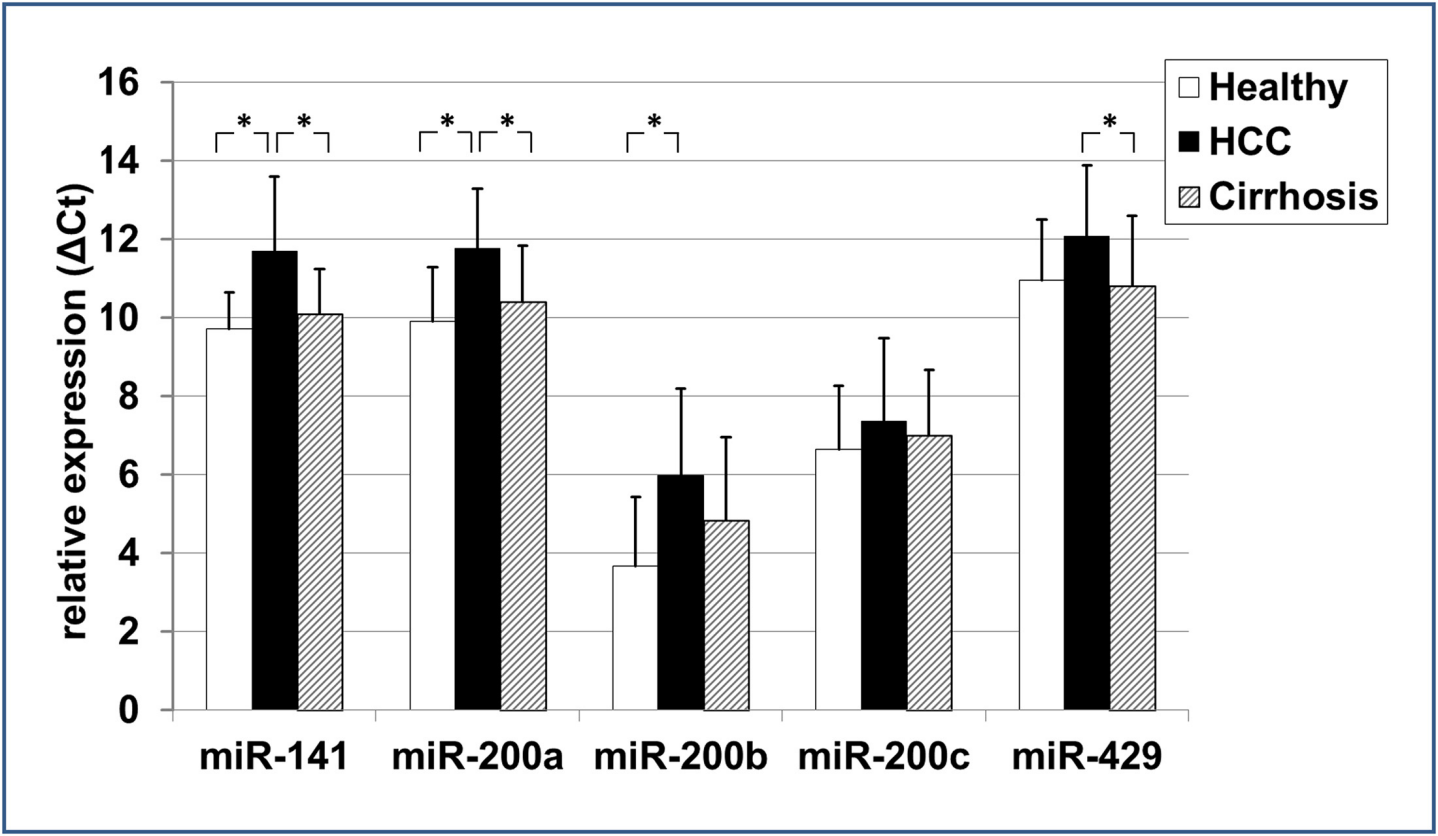
The ΔCt expression level of the five circulating microRNA-200 family members. ΔCt levels are inversely proportional to the amount of target microRNA in the sample. Asterisks indicate to a significant difference of *p*< 0.05, respectively.

**Fig 3:**
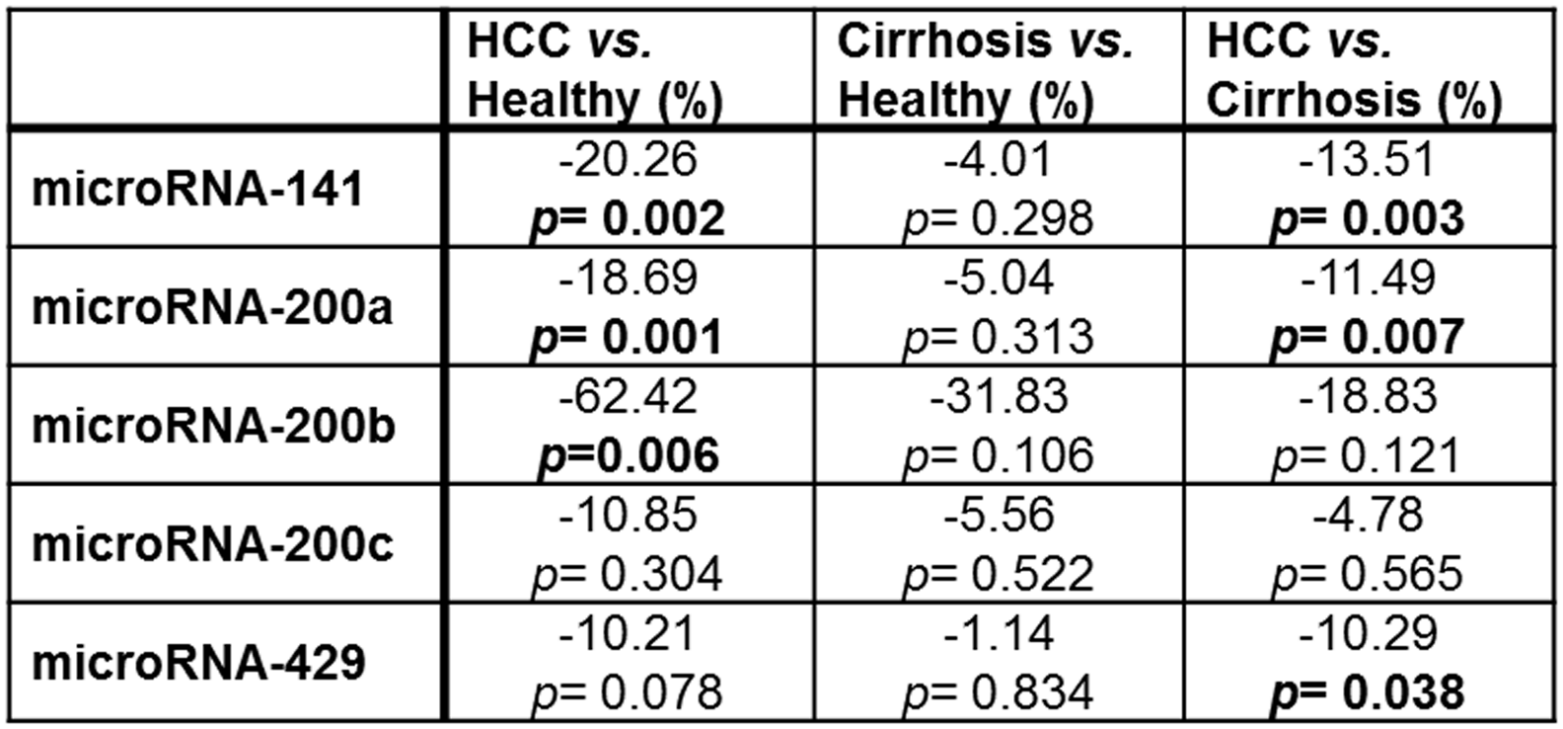
Serum microRNA-200 family level differences between the different study groups. All data are given as median ΔCT level expression differences in % including *p*-value. P<0.05 indicates significance.

The level of circulating microRNA-200 family members in 22 blood serum samples of HCC patients was compared to serum samples of patients with non-cancerous liver cirrhosis (n = 22) and of healthy volunteers (n = 15) (Fig 2). Serum samples of HCC *vs*. healthy liver revealed a significant lower serum concentration of microRNA-141 (-20.26%; *p* = 0.002), microRNA-200a (-18.69%; *p* = 0.001), and microRNA-200b (-62.42%; *p* = 0.006) (Fig 3) in ΔCT analysis. Serum levels of microRNA-141 (-13.51%; *p* = 0.003) and microRNA-200a (-11.49%; *p* = 0.007) were significantly lower in HCC *vs*. non-cancerous liver cirrhosis as well. Significant differences in serum microRNA-200 family concentration were not observed between individuals with healthy and non-cancerous cirrhotic liver.

The same calculation we performed excluding the four HCC patients without cirrhosis and received similar results: Cirrhosis-associated HCC (n = 18) *vs*. healthy liver revealed a significant lower serum concentration of microRNA-141 (-21.28%; *p* = 0.001), microRNA-200a (-19.24%; *p* = 0.001), and microRNA-200b (-62.40%; *p* = 0.005) in ΔCT analysis. Circulating microRNA-141 (-14.24%; *p* = 0.002) and microRNA-200a (-11.90%; *p* = 0.007) were significantly lower in cirrhosis-associated HCC *vs*. non-cancerous liver cirrhosis as well.

**Fig 4:**
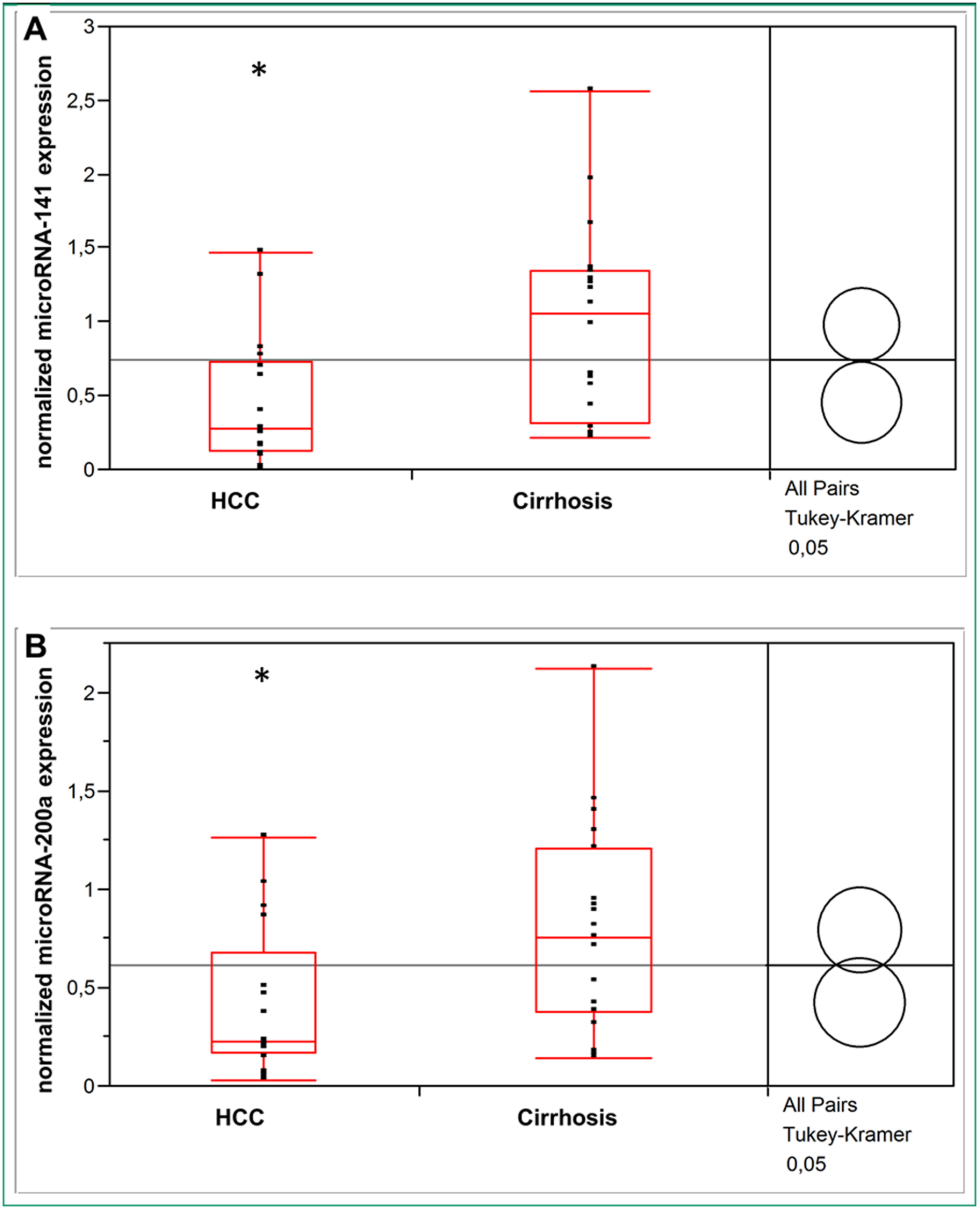
The 2^-ΔΔCt^ expression level of circulating microRNA-141 (A) and microRNA-200a (B) in patients with HCC and non-cancerous liver cirrhosis. Asterisks indicate to a significant difference of *p*< 0.05 versus cirrhosis.

Normalization of circulating microRNA levels in HCC and liver cirrhosis to healthy liver controls by the ΔΔCT method confirmed a significant lower serum concentration of microRNA-141 (*p* = 0.009) and microRNA-200a (*p* = 0.03) in HCC *vs*. non-cancerous cirrhotic liver (Fig 4).

### Evaluation of potential cofactors influencing microRNA-141 and microRNA-200a expression

**Fig 5:**
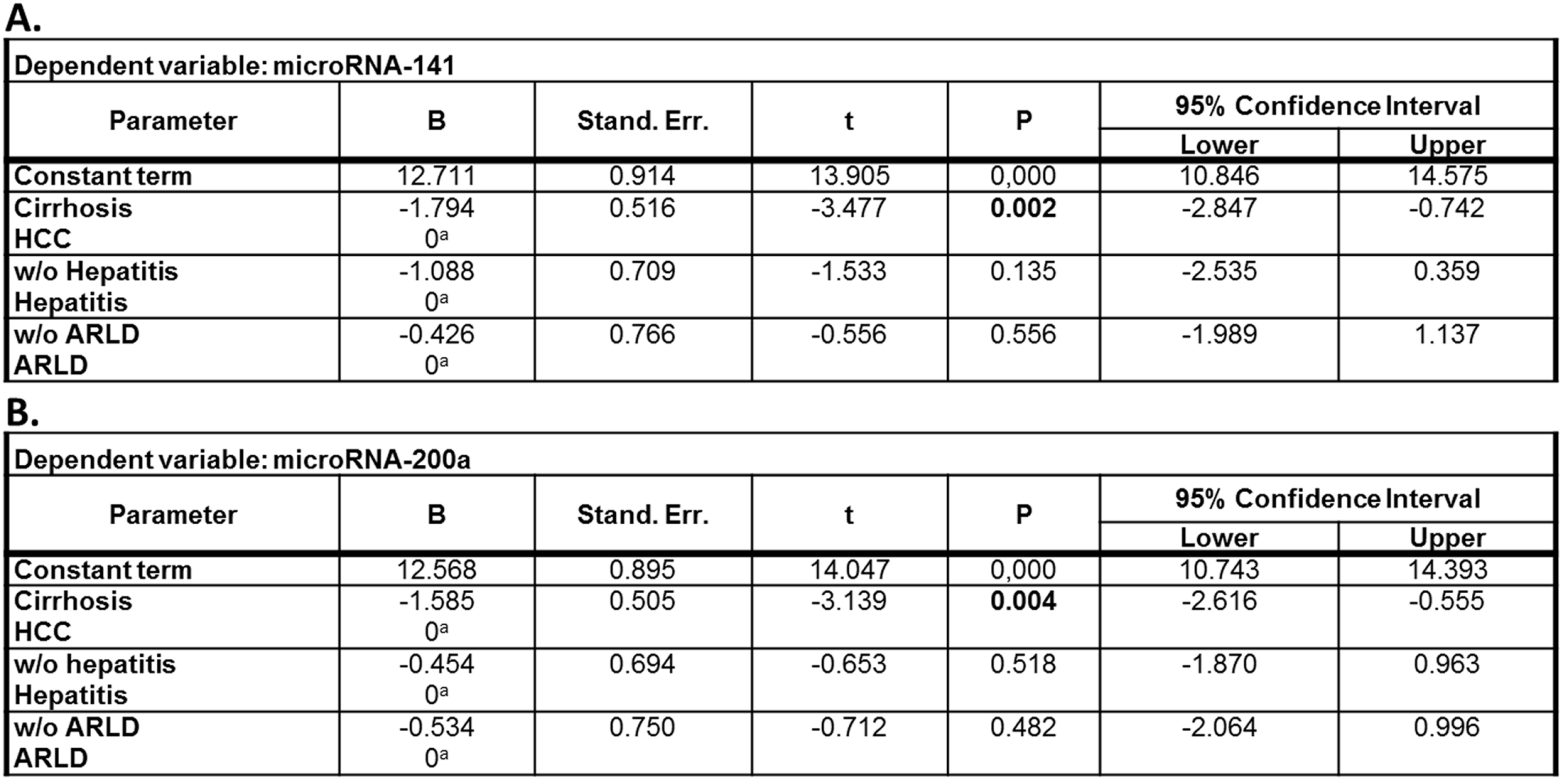
Linear regression analysis of microRNA-141 and microRNA-200a regulating parameters. HCC correlated significantly (p<0.05) with circulating microRNA-141 (A) and microRNA-200a (B) levels. B: regression coefficient B; Stand. Err.: Standard Error; w/o: without; ARLD: alcohol-related liver disease; ^**a**^ This parameter has been set to zero because it is redundant.

Whether other factors besides HCC have an influence on circulating levels of deregulated microRNA-141 and microRNA-200a expression, we further evaluated the statistical relationship between HCC, hepatitis, and alcohol-related liver disease (ARLD) as independent variables and the two deregulated microRNAs as dependent variables by linear regression analysis. As shown in Fig 5, we found that only HCC (*p* = 0.002), but neither hepatitis (*p* = 0.135) nor ARLD (*p* = 0.556) correlated significantly with circulating microRNA-141 and microRNA-200a levels.

**Fig 6:**
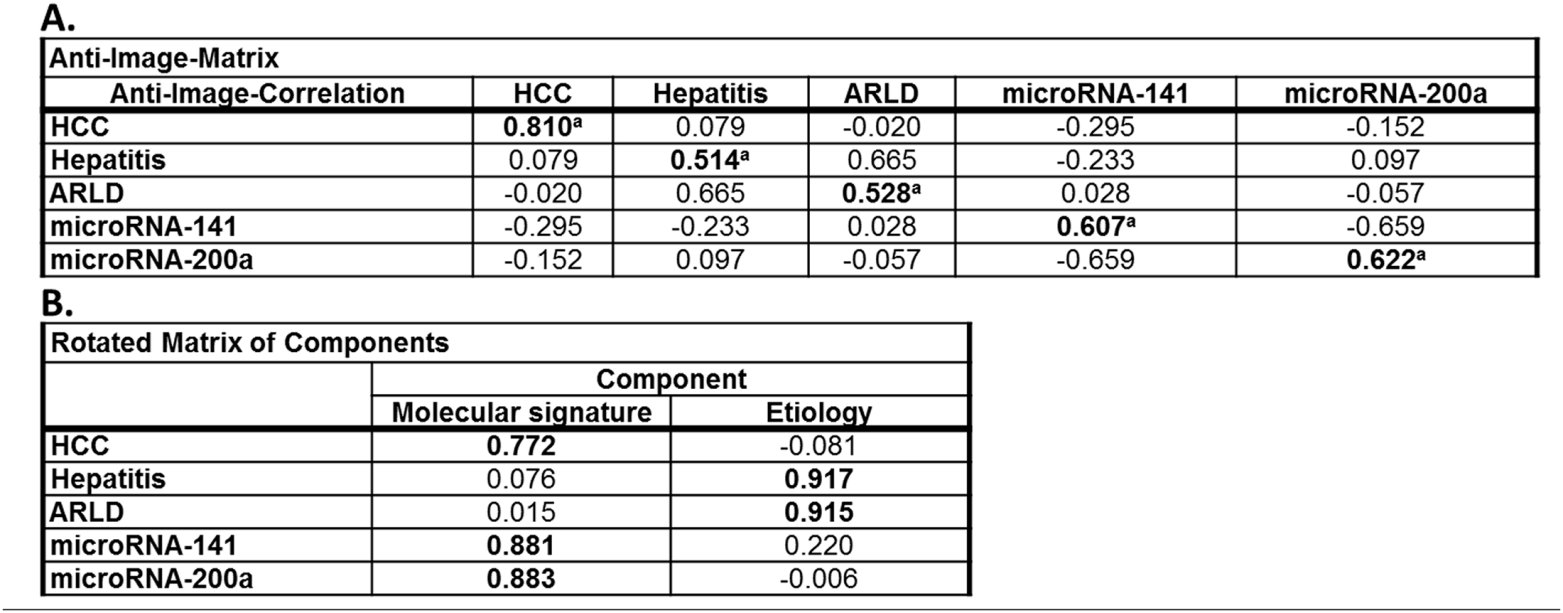
Factorial analysis by anti-image-matrix (A) and Varimax-matrix rotation (B). Sufficient suitability of microRNA expression and etiological parameters with coefficients >0.5 (^a^ suitability criteria). Rotated matrix of components shows a high factorial load by combining microRNA expression and HCC. Factor annotations of initial variables are in bold.

**Fig 7:**
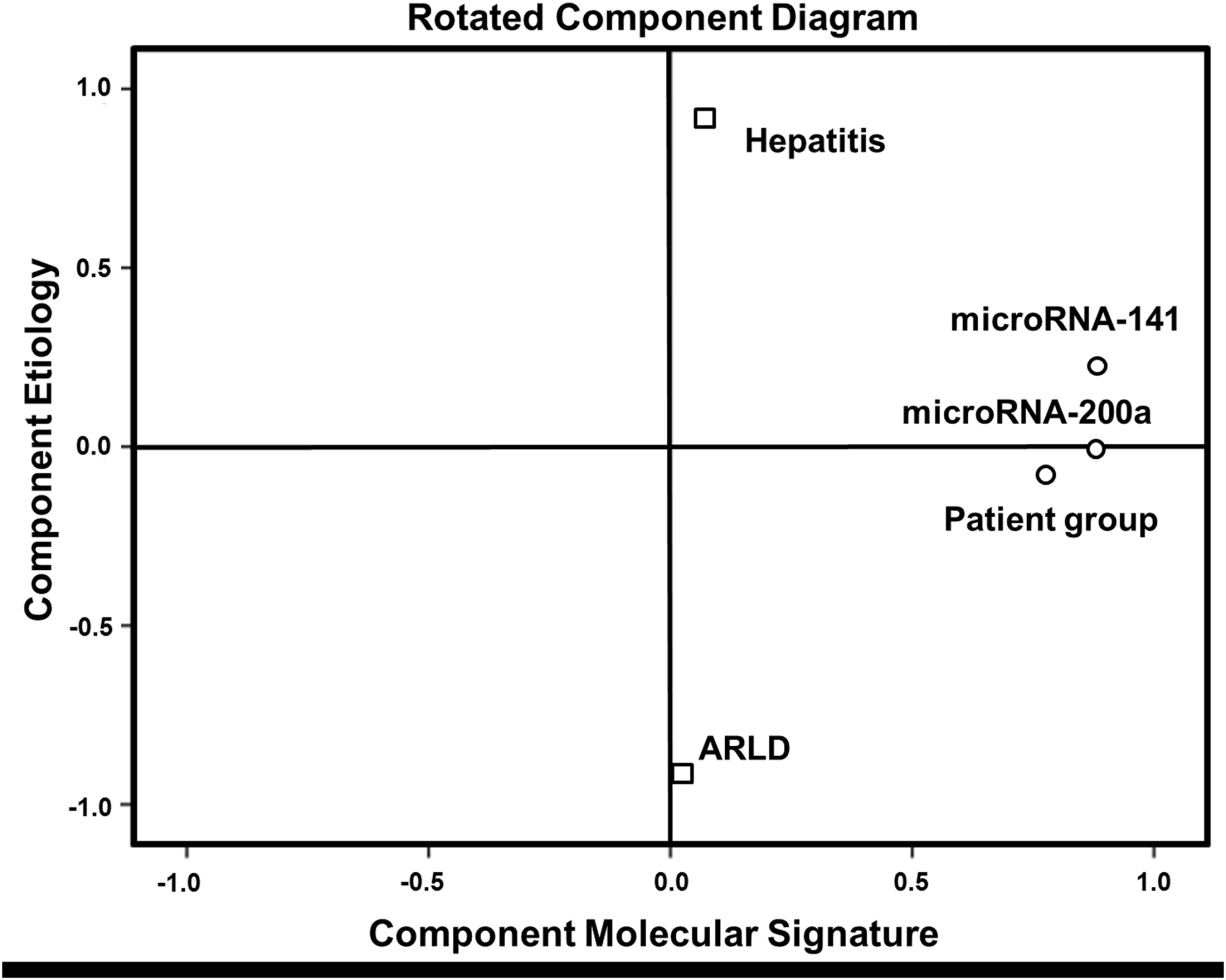
Factorial analysis of molecular signature and etiological variables. Rotated component diagram demonstrates high independence of microRNA-141 and microRNA-200a from hepatitis and alcohol-related liver disease (ARLD) and a high factorial load by combining molecular signature and patient group (HCC, liver cirrhosis).

This regression method investigates for linear dependence of microRNA expression and etiological parameters, but linear regulation of microRNAs cannot be uncritically assumed, we further used factorial analysis for further evaluation. The obtained anti-image-matrix showed sufficient suitability with coefficients >0.5 for all variables (Fig 6A). KMO (0.601) and Bartlett-test (p<0.01) proved applicability of factorial analysis. After primary extraction Kaiser criterion and Scree-plot (not shown) suggested further use of 2 factors that enabled annotation of all variables with high factorial load. These components reflect 1) molecular signature and 2) etiological factors. For improvement of factorial load Varimax-matrix rotation (Kaiser criterion, 3 iterations) was done based on the poor correlation between primary variables. Figs 6B and 7 demonstrate high independence of obtained factor `molecular signature`from the factor `etiology`and high factorial load by combining molecular signature and diagnosis.

### Evaluation of microRNA-141 and microRNA-200a as diagnostic serum markers

**Fig 8:**
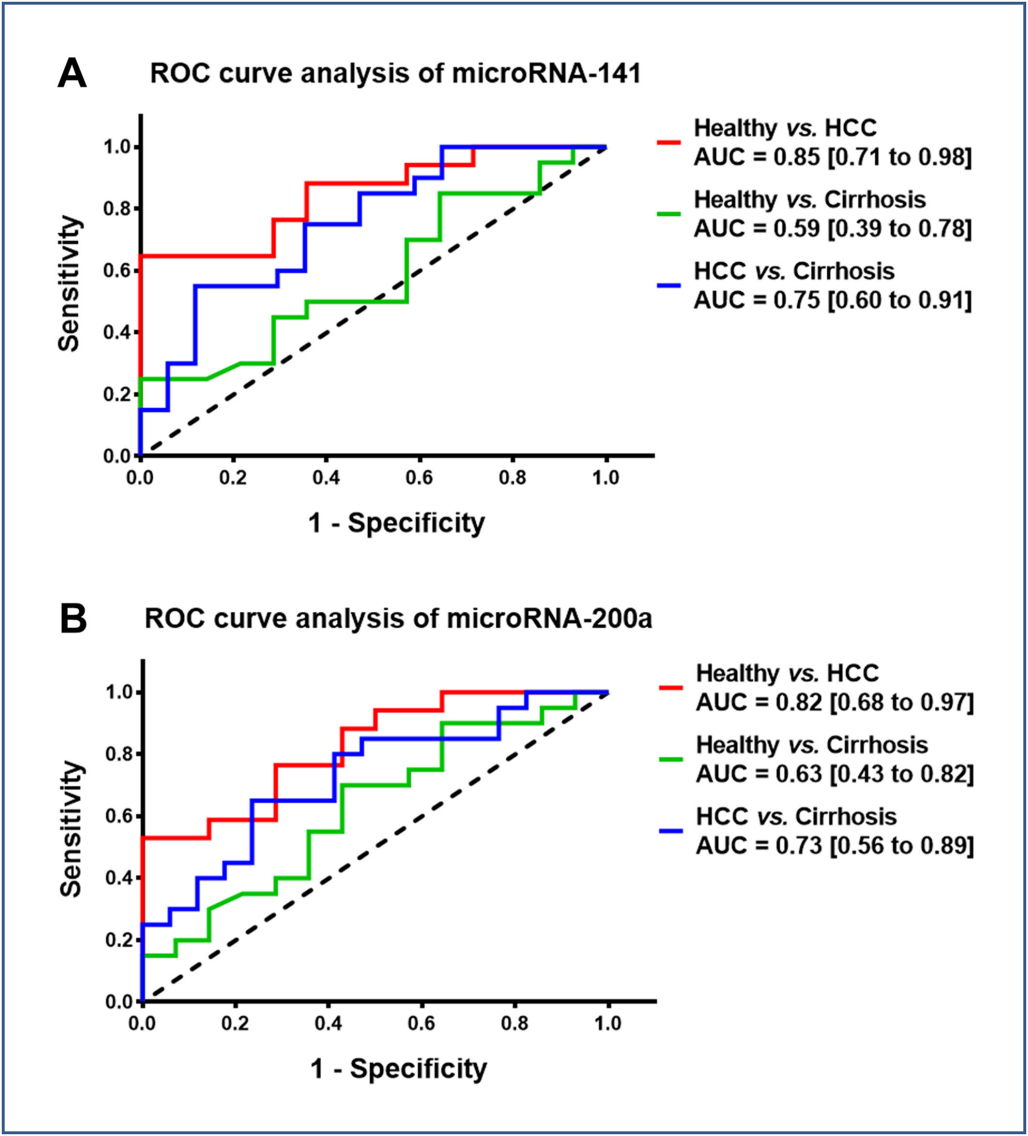
Diagnostic performance of microRNA-141 and microRNA-200a. Receiver operating characteristic (ROC) curve of microRNA-141 (A) and microRNA-200a (B) in three groups (Healthy *vs*. HCC; Healthy *vs*. Cirrhosis; HCC *vs*. Cirrhosis). Area under the curve (AUC) values are presented by the estimate with 95% confidence interval.

Given that circulating microRNA-141 and microRNA-200a levels were significantly lower in patients with cirrhosis-associated HCC, we further evaluated the diagnostic accuracy of plasma microRNA-141 and microRNA-200a as biomarkers to discriminate HCC patients from cirrhosis patients or healthy controls by plotting ROC curves. As shown in Fig 8, we found that microRNA-141 and microRNA-200a could well discriminate patients with cirrhosis-associated HCC from healthy volunteers with AUC values of 0.85 (95% CI: 0.71–0.98; *p* = 0.001) and 0.82 (95% CI: 0.68–0.97; *p* = 0.002), respectively. Additionally, both microRNAs could differentiate between HCC and non-cancerous liver cirrhosis with a fair accuracy of 0.75 (95% CI: 0.60–0.91; *p* = 0.009) and 0.73 (95% CI: 0.56–0.89; *p* = 0.019), respectively. However, the ability of serum microRNA-141 and microRNA-200a in differentiating patients with non-cancerous liver cirrhosis from healthy controls was not satisfactory.

## Discussion

Current diagnostic methods by radiological imaging and AFP serum analysis remain deficient for detecting HCC at early and surgically manageable stages in patients with liver cirrhosis. Thus, novel non-invasive biomarkers are of great clinical value to improve the diagnostic accuracy in early HCC detection.

MicroRNAs have been proved to play a crucial role in human carcinogenesis, including hepatocarcinogenesis, and accumulating experimental evidence indicates that they may act as oncogenes or tumor suppressor genes.[14] Recently, we could show that members of the microRNA‐200 family have potential as diagnostic markers for the detection of HCC in the context of liver cirrhosis, acting as regulators of the epithelial–mesenchymal transition.[10] Over the last years, emerging evidence suggests that circulating serum microRNAs are stable and thus can serve as non-invasive diagnostic biomarkers for different gastrointestinal carcinomas.[15–22] Therefore, in the present study we investigated the diagnostic value of the microRNA‐200 family as circulating biomarker in clinical blood serum samples of HCC patients. In accordance with our liver tissue study, low serum microRNA-141 and microRNA-200a expression correlated significantly with HCC prevalence. In HCC serum samples no significant differences were observed for the other microRNA-200 family members. Factor analysis and linear regression analysis revealed that HCC tissue type, but neither hepatitis nor ARLD are significantly related to circulating microRNA-141 and microRNA-200a levels and the molecular components are sufficient to explain the observed variances. This was confirmed by analysis of the results neglecting the non-cirrhosis HCC patients. Combining these results with the second analysis without non-cirrhosis patients, etiological factors do not have significant importance for the observed molecular data. Additionally, serum microRNA-141 and microRNA-200a were able to differentiate between HCC and healthy liver with a good accuracy. However, the only fair accuracy for distinguishing the HCC patients from the non-cancerous cirrhotic controls may have been due to the limited sample size. Future large scale investigations are requested to also evaluate the potential influence of intraindividual variations, such as circadian rhythm.[23] In order to limit these potential influences in our study standardized sample handling was applied. Consistent with our results, Liu *et al*. described microRNA-141 as a tumor suppressor which may serve as an independent prognostic factor in HCC.[24] Downregulation of circulating microRNA-200a was recently discovered in hepatitis B virus-positive small HCC.[25] For a sufficient evaluation of survival data and the role of the microRNA-200 family as potential prognostic marker the inhomogeneity within our study groups and the resulting limited statistical power were not satisfying. The value of microRNA‐200 family members as prognostic circulating biomarkers for HCC in liver cirrhosis remains undefined and larger prospective studies need to investigate its prognostic value for HCC.

The underlying mechanism responsible for the decreased expression of intracellular and circulating microRNA-141 and -200a in HCC remains unknown. Interestingly, according to the similarity of their seed sequence two different clusters can be identified: microRNA-200b/-200c/-429 and microRNA-200a/-141, which are differentiated by a single nucleotide change.[26]

In conclusion, our study indicates that circulating microRNA-200 family members are significantly differentially detectable in patients with HCC, suggesting their potential diagnostic value as non-invasive serum markers. However, additional larger sample trials for selecting appropriate cut-off points are required to confirm our findings. Furthermore, questions about induction of microRNA-200 family members and their individual function in physiological development and carcinogenesis remain to be answered. The clarification of these points might lead to new therapeutic approaches for diseases such as liver cirrhosis and HCC.
